# Neuroinflammation in retinitis pigmentosa: Therapies targeting the innate immune system

**DOI:** 10.3389/fimmu.2022.1059947

**Published:** 2022-10-27

**Authors:** Ling Zhao, Chen Hou, Naihong Yan

**Affiliations:** ^1^Research Laboratory of Ophthalmology, West China Hospital, Sichuan University, Chengdu, China; ^2^Department of Ophthalmology, West China Hospital, Sichuan University, Chengdu, China

**Keywords:** retinal inflammation, innate immune, gut microbiome, trained immunity, epigenetic modification, retinitis pigmentosa

## Abstract

Retinitis pigmentosa (RP) is an important cause of irreversible blindness worldwide and lacks effective treatment strategies. Although mutations are the primary cause of RP, research over the past decades has shown that neuroinflammation is an important cause of RP progression. Due to the abnormal activation of immunity, continuous sterile inflammation results in neuron loss and structural destruction. Therapies targeting inflammation have shown their potential to attenuate photoreceptor degeneration in preclinical models. Regardless of variations in genetic background, inflammatory modulation is emerging as an important role in the treatment of RP. We summarize the evidence for the role of inflammation in RP and mention therapeutic strategies where available, focusing on the modulation of innate immune signals, including TNFα signaling, TLR signaling, NLRP3 inflammasome activation, chemokine signaling and JAK/STAT signaling. In addition, we describe epigenetic regulation, the gut microbiome and herbal agents as prospective treatment strategies for RP in recent advances.

## 1 Introduction

### 1.1 Retinal inflammation and RP

Retinitis pigmentosa (RP) is a category of inherited retinal dystrophies marked by vision loss and, ultimately, blindness. More than 3000 mutations in over 80 distinct genes or loci have been identified as causes of non-syndromic RP (1, 2). These mutations can be transmitted in an autosomal-dominant, autosomal-recessive, or X-linked manner. There are also syndromic forms of RP, such as Usher syndrome and Bardet-Biedl syndrome (3). The prevalence of RP is reported to be 1/3,000 to 1/5,000 (https://www.orpha.net/consor/cgi-bin/index.php?lng=EN). Early in the progression of RP, patients experience night blindness and difficulty with dark adaptation. They gradually lose peripheral vision and develop tunnel vision as the disease advances, indicating the loss of rod function. Cone involvement contributes to visual acuity decline over time, and finally, typically in middle age, central vision loss occurs (2). Due to its clinical and genetic heterogeneity, RP has limited therapeutic options. It has long been recognized that inflammation and immune responses are associated with RP, and this theme has recently gained increasing attention (4–6). Greater understanding of the molecular processes driving RP inflammation is expected to provide new therapeutic approaches independent of the genetic background.

Inflammation activation is a prominent feature of RP. In RP, inflammation is characterized by activation of the innate immune system, including dysfunction of the immune barrier, activation and infiltration of immune cells, and upregulation of topical and peripheral inflammatory factors. Bone-spicule pigmentation, attenuated retinal vessels and waxy pallor of the optic disc are typical clinical manifestations of RP. In addition, inflammatory cells are commonly observed in the vitreous due to the collapse of the blood−retina barrier (BRB). Higher cell density correlates with younger age and impaired visual function (6). In addition, it has been reported that increased aqueous flare in RP patients correlates closely with visual function and the extent of global retinal degeneration (7–11). Aqueous flare is generally seen in individuals with inflammatory ocular disorders, indicating deficits of the blood–aqueous barrier and inflammatory protein/cell leakage (12, 13). BRB disruption begins early in the disease. Prior to the infiltration of inflammatory cells and photoreceptor (PR) starvation, the tight junctions of the retinal pigment epithelium (RPE) and the retinal vasculature become leaky, thereby promoting the formation of an inflammatory milieu and the degeneration of PRs (14–17).

Microglia are essential components of the retinal innate immune system and play a pivotal role in retinal inflammatory responses. Gupta et al. reported that microglial activation is engaged in human RP. With thinning of the PR layer, microglia were observed to infiltrate degenerative foci; these microglia were enlarged amoeboid cells containing rhodopsin-positive cytoplasmic inclusions (18). Microglia promote retinal inflammation *via* infiltration, phagocytosis, and secretion of proinflammatory mediators, whereas genetic ablation or inhibition of microglial phagocytosis ameliorates PR degeneration in RP model mice (19).

Several studies (6, 20–22) have reported elevated levels of inflammatory factors in serum, vitreous, and aqueous humor, indicating a proactive inflammatory response in RP patients. Immunologic disorders observed in patients with RP are summarized in a previous work (23).

Animal models are essential for the study of RP (24, 25). Activation of the immune system has been detected in various rodent RP animal models (26–28). Due to this similarity, animal models are indispensable for clarifying the pathogenesis of RP and developing treatments. Clinically, synthetic corticosteroids with potent anti-inflammatory and immunosuppressive properties are used in treatments for RP-related cystoid macular edema (29). In RP models, it also works. In RCS and rhodopsin mutant model S334ter-4 rats, fluocinolone acetonide treatment markedly protects PR from degeneration and suppresses microglial activity (30, 31). In combination with polyamidoamine dendrimers, fluocinolone acetonide selectively targets the microglia localized in the outer retina where degeneration is ongoing (32). Dexamethasone administration to rd10 mice reduces retinal inflammation, restores cone structure and function, and preserves RPE integrity by preserving ZO-1 density (15, 33).

### 1.2 Microglia and Müller glia in RP retinas

Microglial activation is a sign of neuroinflammation. Retinal resident microglia arise from yolk sac erythromyeloid progenitors, comprising 85% of total retina macrophages (34–36). They colonize the developing retina during embryogenesis, shape the retina by secreting neurotrophic factors, engulf and eliminate unwanted neurons and synapses, and engage in vascular development of the eye (37–39). In postnatal retinas, microglia are maintained throughout life independent of circulating monocytes and by self-renewal (34, 40, 41).

Microglia express a variety of receptors (e.g., CX3CR1, TLRs, IL-1R, and TNFR) that allow them to detect environmental changes and initiate the inflammatory signal cascade (42). Microglia activation induces a robust inflammatory response, including the release of proinflammatory factors, phagocytosis, and inflammatory cell recruitment. Excessive microglial phagocytosis contributes to local inflammation and neurodegeneration (43, 44).

Classically, activated microglia were categorized into two groups: M1 (classically activated) and M2 (alternatively activated), with the belief that M1 microglia secrete proinflammatory factors such as TNFα, IL-1β, IL-6, and inducible nitric oxide synthase (iNOS) that fuel inflammation, whereas M2 microglia produce anti-inflammatory cytokines (e.g., IL-4, IL-10, IL-13, IL-18) that are beneficial for damage repair (45). Recent research, however, suggests that the microglial phenotype varies in response to environmental changes (46).

In healthy retinas, microglia tile the inner and outer plexiform layers without overlapping (37), where they are ramified cells responsible for immune surveillance and maintenance of synaptic structure and transmission (47–50). In response to insults, microglia rapidly change into an amoeboid appearance and migrate into lesion areas, removing dead/dying neurons and neuronal debris while concurrently releasing proinflammatory factors as well as protective cytokines and trophic factors to repair damage and restore homeostasis (51), after which microglia recover to the “resting” state; this process usually results in minimal retinal remodeling.

In RP retinas, initial mutation-driven PR degeneration increases extracellular signal molecules (e.g., ATP, HSPs, HMGB1, DNA, and many others) termed damage-associated molecular patterns (DAMPs) (52–56). The “eat-me” signal, phosphatidylserine, appears on stressed rods (19). Microglia proliferate and infiltrate the PR layer and subretinal space, where they function as reactive phagocytes, phagocytose dead and stressed PRs, secrete proinflammatory cytokines (e.g., TNFα and IL-1β) and chemokines (e.g., CCL2 and RANTES), and recruit infiltrating immune cells (19, 24, 57, 58). Due to this mutant genetic background, however, microglial activation persists, and the continuous production of inflammatory and cytotoxic factors exacerbates PR loss until the late stage, at which point PRs mostly die and the retinal structure is severely damaged (59).

Müller glia are another group of retinal cells engaged in degeneration. Müller glia are retinal macroglia that provide homeostasis, metabolism, and functional support for neurons (60). Depending on the severity, the Müller glial response to injury refers to reactive gliosis accompanied by Müller proliferation or not. Reactive gliosis can be beneficial because it releases protective factors such as neurotrophic factors, whereas prolonged gliosis is detrimental and generally results in neurodegeneration (61). Müller glia are potential modulators of retinal inflammation. Upon BRB disruption, Müller glia compensate for RPE deficiency by sealing the leaky choroid and inducing claudin-5 expression (14). Müller glia share characteristics with immune cells. Müller glia express multiple cytokine receptors and are a major source of cytokines and inflammatory factors (62). Proteomic evidence supports the capacity of Müller glia for antigen presentation and inflammatory signaling transduction in response to immune stimulation (63, 64). Müller glia contribute to the phagocytic clearance of dead PRs (65). Moreover, the interaction between Müller glia and microglia modulates retinal inflammation and degeneration (66–68).

As the predominant glial population of the retina, Müller glia are abundant and widely distributed. Müller glia traverse the thickness of the neuroretina structurally, allowing them to keep touch with all types of retinal cells. Due to its neurotrophic function and regeneration potential, the Müller cell has been studied in a variety of degenerative retinal disorders (69). In actuality, Müller glia are also intimately linked to retinal inflammation. For more information about how Müller glia interact with the innate immune system and monitor retinal inflammation, we refer the reader to this article (70).

**Figure 1:**
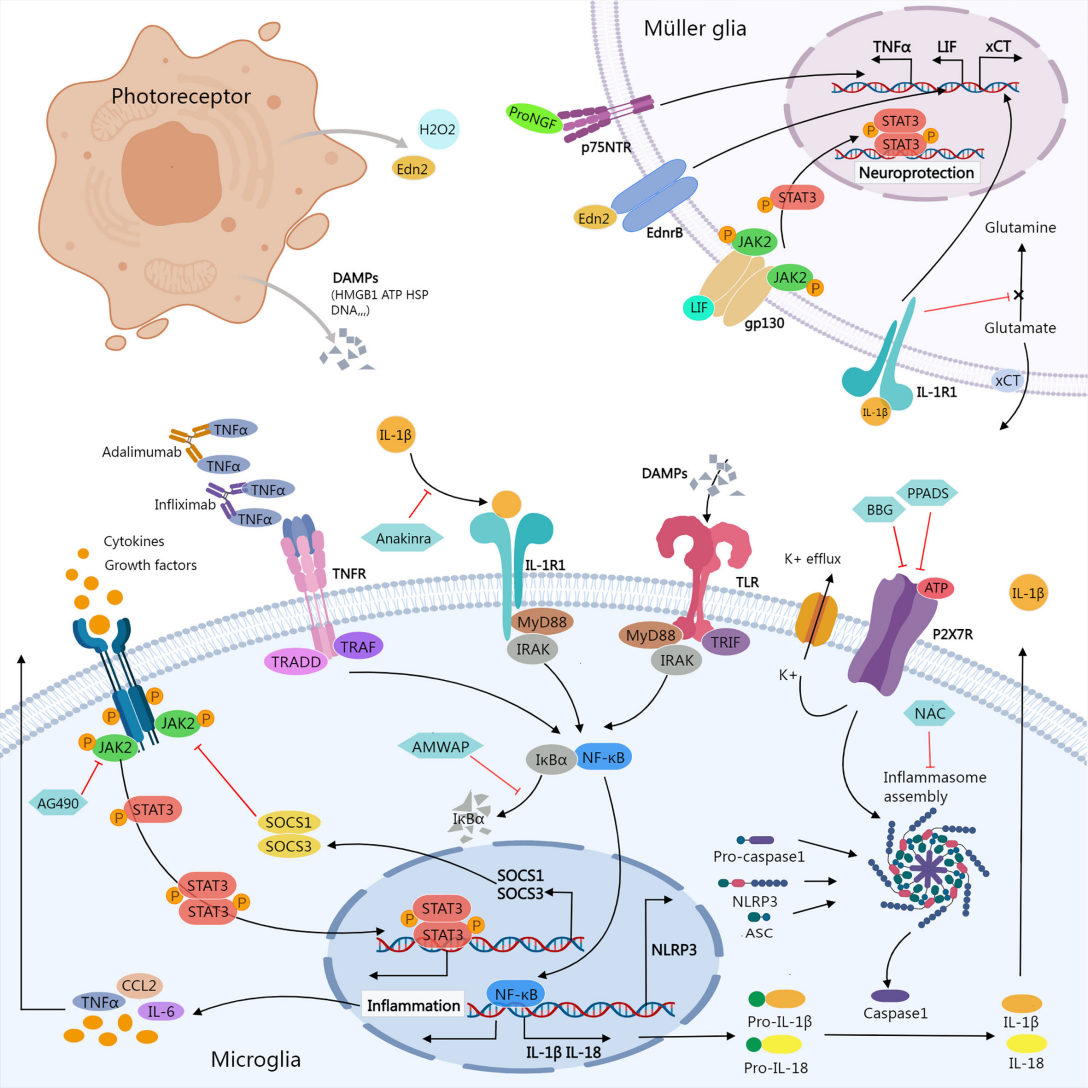
Inflammatory signals between photoreceptor, microglia, and Müller glia. Damaged or dying photoreceptors release signal molecules that stimulate the activation of microglia and Müller glia. In Müller glia, activation of NFGR promotes TNFα production; Edn2 binds to Ednrb promotes LIF transcription; LIF binding to gp130 activates the JAK2/STAT3 pathway, which promotes Müller glial neuroprotection; IL-1β binding to IL-1R1 interferes with glutamate conversion into glutamine, resulting in elevated extracellular glutamate concentration. In microglia, activation of JAK2/STAT3 signaling stimulates the release of inflammatory molecules. SOCS1/SOCS3 act as negative feedback factors of JAK/STAT pathway; AG490 specifically inhibits JAK2. Ligands-binding to TNFR, IL-1R, and TLR stimulates NF-κB signaling cascades, promoting transcription of inflammatory genes including NLRP3, pro-IL-1β, and pro-IL-18; ATP stimulates K+ efflux *via* P2X7R, promoting NLRP3 inflammasome activation and the release of active caspase-1, mature IL-1β, and IL-18. Biological agents like infliximab and adalimumab provide neuroprotection by neutralizing TNFα. Anakinra blocks the biologic activity of IL-1β. AMWAP prevents NF-κB translocating into the nucleus by inhibiting IκBα degradation. NAC suppresses the activation of NLRP3. BBG and PPADS reduce the activation of the NLRP3 inflammasome *via* inhibiting P2X7R signaling. AMWAP, activated microglia/macrophage WAP domain protein; ASC, apoptosis-associated speck-like protein containing a CARD; ATP, adenosine triphosphate; BBG, Brilliant Blue G; CCL2, C-C Motif Chemokine Ligand 2; DAMPs, damage-associated molecular patterns; Edn2, endothelin 2; Ednrb, endothelin receptor B; HMGB1, High-mobility group box-1; HSP, heat shock protein; JAK, janus kinase; LIF, leukemia inhibitory factor; MyD88, myeloid differentiation primary response 88; NAC, N-acetylcysteine; PPADS, pyridoxal-phosphate-6-azophenyl-2’,4’-disulfonic acid; proNGF, pro-nerve growth factor; p75NTR, p75 neurotrophin receptor; STAT, signal transducer and activator of transcription; TNFR, Tumor necrosis factor receptor; TRADD, TNFR1-associated death domain protein; TRAF, TNF receptor associated factor; TLR, Toll-like receptors; TRIF, TIR-domain-containing adaptor-inducing interferon-β; xCT, core subunit of the cystine/glutamate transporter system xc-; IRAK, Interleukin-1 receptor-associated kinase.

Complicated mechanisms are involved in the regulation of retinal inflammation in RP. Microglia and Müller glia are major cellular populations that express and modulate these signaling pathways (**Figure 1**). Here, we review treatment strategies from the perspective of inflammation management (**Table 1**), focus on molecular mechanisms related to immunomodulation, and discuss new findings regarding epigenetic modification and the gut microbiome as novel therapies for RP.

**Table 1:** Treatments for retinitis pigmentosa and related mechanisms.

Mechanism	Approach	Gene/molecule/agent	Effect	Model	Ref.
TNFα signal	Genetic KD TrkC antagonism	TrkC.T1 KB1368	Suppress p-ERK activation and TNFα production	RHOP347S mouse	(71)
	p75NTR antagonism	THX-B	Inhibit reactive gliosis and TNFα secretion	Rd10, RHOP347S mouse	(72)
	Genetic KD	*Tnfα*	Decrease proinflammatory factors (IL-1β, IL-6, IL-17, RANTES, CCL2) as well anti-inflammatory factor (IL-10 and IL-13)	T17MRHO mouse	(73)
	TNFα blockade	Infliximab	Decrease *caspase-3* activation and reactive gliosis	Zaprinast-induced degeneration of porcine retina	(74)
		Adalimumab	Decrease PARP activation, microglia activation and NLRP3 inflammasome activation	Rd10 mouse	(25, 75)
TLR signal	Genetic KO	*Tlr2*	Suppress microglial activation and infiltration	Rd10, P23H mouse	(76)
	Genetic KO	*Tlr4*	Reduce CCL2 expression, microglia activation and gliosis	LD *Abca4*-/- *Rdh8*-/- mouse	(77)
	Microglia inhibition	Minocycline	Suppress microglial activation and migration	P23H-1, RCS rat, *Prph2* ^Rd2/Rd2^ mouse	(78, 79)
		Minocycline	Decrease microglia activation and proinflammatory gene transduction	LD mouse retina	(80, 81)
		Minocycline	Decrease microglia activation and proinflammatory molecule expression	Rd10 mouse	(82)
	Genetic KO	*Myd88*	Reduce chemokine (CCL2, CCL4, CCL7 and CXCL10) expression and microglial activation	Rd1 mouse	(83)
	MyD88 inhibition	MyD88 inhibitor peptide	Suppress microglia infiltration, increase neuroprotective microglia, expression of MCP-1, IL-27 and crystalline	Rd10 mouse	(84, 85)
	AMWAP supplement	AMWAP	Blockade TLR-mediated NF-κB activation	661W cell-microglia co-culture	(86)
NLRP3 signal	NLRP3 inhibition	N-acetylcysteine	Decrease NLRP3 expression and microglial infiltration	Rd10 mouse, P23H rat	(24, 87)
	P2X7R blockade	PPADS	Promote photoreceptor survival	Rd1 mouse	(88)
		BBG	Decrease inflammasome components (NLRP3, cleaved caspase-1 and mature IL-1β proteins)	P23H rat	(87)
	IL-1β blockade	Anakinra	Reduce photoreceptor apoptosis	Rd10 mouse	(19)
CX3CL1/CX3CR1	CX3CL1 supplement	CX3CL1	Decrease microglial infiltration, phagocytosis and activation	Rd10 mouse	(89)
		AAV8-sCX3CL1	Improve cone survival	Rd1, rd10 and rhodopsin null mouse	(90)
		Norgestrel	Upregulate CX3CL1/CX3CR1 signal	Rd10 mouse	(91, 92)
CCL2/CCR2	CCR2 inhibition	Lecithin-bound iodine Minocycline	Suppress CCR2 positive macrophage invasion	*Mertk* mouse	(93, 94)
	Genetic KO	*Ccr2*	Reduce apoptosis	Rd10	(95)
		*Ccl2/Ccl3*	Reduce retinal inflammation	*Mertk* mouse	(96)
	Genetic KD	CCL2 siRNA	Decrease monocyte/microglia infiltration	LD rat retina	(97)
JAK/STAT pathway	JAK/STAT inhibition	AG490	Improve PR survival	LD mouse retina	(98)
		OECs transplantation	Inhibit JAK2/STAT3 activity and increase SOCS3	RCS rat	(99)
	JAK/STAT activation	pMSC-RPCs transplantation	Activate JAK/STAT, improve retinal structure and function	Rd12	(100)
	CNTF supplement	rAAV2/2-hCNTF	Upregulate STAT3, SOCS3, SOCS5 and complement factor (C3, C4a, Cfb) expression	Rhodopsin null mouse	(101)
		LV-hCNTF	Stimulate expression of LIF, Edn2; activate gp130/JAK/STAT	Rds/peripherin P216L transgenic mice	(102)
		LIF	Activate STAT3, improves PR survival	LD mouse retina	(103)
Epigenetic modification	HDACi	Trichostatin A	Decrease activity of PARP, preserve cone survival	Rd1, rd10 mouse	(104, 105)
		Tubastatin A	Improve cone survival, alter expression about ubiquitin-proteasome, phototransduction, metabolism and phagosome	Zebrafish, *atp6v0e1*(-/-)zebrafish, rd10 mouse	(106, 107)
		Romidepsin	Inhibit transcription of inflammatory genes and inflammation	Rd10 mouse	(108)
		Valproic acid	Protection varies with genotype		(109, 110)
	BET inhibition	JQ1	Suppress microglial proliferation, migration, and cytokine production	Rd10 mouse	(111)
	LSD1 inhibition	Tranylcypromine; GSK2879552	Inhibit transcription of inflammatory genes and inflammation	Rd10 mouse	(108)
	H3K27me3 inhibition	DZNep	Improve PR survival	Rd1 mouse	(112)
	miRNA inhibition	AAV-miRNA modulator of miR-6937	Improve ONL thickness and ERG response	Rd10 mouse	(113)
	miRNA supplement	AAV- miR-204	Suppress microglia activation	RHO-P347S mouse	(114)
Herbal agent		Curcumin	Inhibit microglia activation and expression of CCL2, TIMP-1, improves retinal morphology	Rd1 mouse, P23H rat	(115, 116)
		Lyceum barbarum polysaccharides	Inhibit NF-κB and HIF-1α expression	Rd1, rd10	(117, 118)
		Zeaxanthin dipalmitate	Inhibit STAT3, CCL2, MAPK pathways	Rd10 mouse	(119)
		Saffron	P2X7R signaling blockade, decrease vascular disruption	ATP-induced PR death, P23H rat	(120, 121)
		Resveratrol	Downregulate microglial migratory, phagocytic, and proinflammatory cytokine production	Microglia-mediated 661W death	(122)
		JC19	Improve PR survival, sirtuin1 activation may be the protective mechanism	Rd10 mouse	(123)

THX-B, (1,3-diisopropyl-1-[2-(1,3-dimethyl-2,6-dioxo-1,2,3,6-tetrahydro_purin-7-yl)-acetyl]-urea; KD, knock down; KO, knock out; LD, light damage; MyD88, myeloid differentiation factor 88; AMWAP, activated microglia/macrophage whey acidic protein; PPADS, pyridoxal-phosphate-6-azophenyl-2’,4’-disulfonic acid; BBG, Brilliant Blue G; AAV, adeno-associated virus; siRNA, small interfering RNA; PR, photoreceptor; OECs, olfactory ensheathing cells; SOCS, suppressor of cytokine signaling; pMSC-RPCs, primitive mesenchymal stem cell-derived retinal progenitor cells; rAAV2/2, recombinant adeno-associated virus serotype 2; LV, lentiviral vector; LIF, leukemia inhibitory factor; Edn2, endothelin2; HDACi, histone deacetylase inhibition; BET, bromodomain and extraterminal domain; LSD1, lysine demethylase 1; miRNA, microRNA; ONL, outer nuclear layer; ERG, electroretinogram; TIMP-1, tissue inhibitor of metalloproteinases 1.

## 2 Mechanisms related to inflammation in RP

### 2.1 TNFα signaling

Tumor necrosis factor α (TNFα) is a strong proinflammatory cytokine that plays vital roles in immune modulation, cell proliferation, differentiation, and apoptosis. TNFα is produced predominantly by T and innate immune cells and is initially synthesized as transmembrane protein (tmTNFα), a precursor that requires proteolytic cleavage by TNFα-converting enzyme (also ADAM17) to release a soluble form (sTNFα) (124). Both tmTNFα and sTNFα are implicated in the inflammatory response.

TNFα initiates a signal cascade by binding to its receptors, TNFR1 and TNFR2. TNFR1 is activated by both tmTNFα and sTNFα, whereas TNFR2 is proposed to be fully activated primarily by tmTNFα (125). Ligand binding to TNFR1 recruits the adaptor molecule TNFR1-associated death domain protein, which then leads to the assembly of several signaling complexes known as complexes I, IIa, IIb, and IIc. Complex I formation stimulates nuclear factor kappa B (NF-κB) and mitogen-activated protein kinases (MAPKs). Complex IIa and IIb assembly activates caspase-8 and facilitates apoptosis, and complex IIc formation activates the mixed lineage kinase domain-like protein and induces necroptosis and inflammation. TNFR2 stimulation activates NF-κB, MAPKs, and protein kinase B (125).

TNFα is postulated to participate in the pathogenesis of RP (74). TNFα and TNFR expression levels are elevated in the retina of RP models and in the aqueous humor of RP patients (24, 74, 126–128); microglia (129) and Müller glia (130, 131) are the primary cellular sources of TNFα. TNFα signaling has been found to mediate PR death *via* RIP1/3-related necrosis and caspase3/7-dependent apoptosis, in addition to triggering proinflammatory signaling in the RP retina (73, 126).

#### 2.1.1 NGF receptor and TNFα production

Increased TNFα expression in the retina is linked to nerve growth factor (NGF) receptor activation. Müller glia in rhodopsin mutant RP model RHOP347S mice upregulate the expression of TrkC. T1, a truncated TrkC receptor isoform, and its ligand NT-3. TrkC.T1 increases local TNFα production by activating MAPK/Erk, ultimately leading to PR death. This process can be reversed by genetic knockdown of TrkC.T1, TrkC antagonism, or MAPK/Erk inhibition (71). Similarly, TrkC.T1 knockout (KO) and TrkC inhibition increased retinal ganglion cell survival in a mouse model of glaucoma by reducing TNFα production (132), implying that TrkC.T1 is upstream of TNFα.

It has been reported that microglia-derived proNGF facilitates PR death *via* p75NTR (133), proNGF binding to p75NTR in Müller glia induces robust expression of TNFα and TNFα-dependent neuron death in rodent retina (131, 134), the expression levels of proNGF and p75NTR are increased in the retina of rd10 at early degenerative stages, pharmacological antagonism of p75NTR with THX-B ((1,3-diisopropyl-1-[2-(1,3-dimethyl-2,6-dioxo-1,2,3,6tetrahydro-purin-7-yl)-acetyl]-urea)) affords neuroprotection to PRs, and the treatment also mediates reduction of TNFα production, microglial activation, and reactive gliosis (72).

#### 2.1.2 TNFα inhibition

TNFα knockdown in the T17M rhodopsin mutant mouse model reduces PR death and PR-related functional loss, and this neuroprotective effect is associated with reductions in proinflammatory cytokines (IL-1β, IL-6, IL-17, RANTES) and chemokines (CCL2) (73).

Infliximab and adalimumab are biological TNFα inhibitors approved for treating inflammatory disorders such as Crohn’s disease, ulcerative colitis, rheumatoid arthritis, plaque psoriasis, and uveitis (125, 135). By lowering the expression of TNFR1 and caspase-3 activity, infliximab alleviated retinal degeneration induced by PDE6 inhibition in cultured porcine retina (74, 127). Adalimumab administered intraperitoneally or topically improved PR survival while decreasing microglial activation and reactive gliosis in the rd10 retina. Inhibition of PARP and RIPK signaling, as well as NLRP3 inflammasome assembly, are mechanisms involved in this protective response (25, 75).

#### 2.1.3 Protective effects of TNF signaling

Notably, TNFα KO retinas tend to express both pro- and anti-inflammatory factors at reduced levels when compared with controls (73), implying that removing TNFα would also damage the immune system’s defenses. Recent work by Kuhn et al. established that TNFα, in collaboration with TNFR1, TNFR2, and p75NTR, induces signals that are indispensable for neural development and that disturbances to TNFR family signaling result in unhealthy axonal development (136).

ADAM17 regulates the expression of TNFα as well as the receptor TNFR (124). Muliyil et al. reported that ADAM17 and soluble TNF mediate a novel cytoprotective pathway in *Drosophila*. Loss of ADAM17 or TNF/TNFR signaling drives the accumulation of lipid droplets and degeneration in the *Drosophila* retina, whereas restoration of ADAM17 or TNF/TNFR in glia is sufficient to rescue the degeneration phenotype. TNF and TNFR are explicitly needed in glia; loss of either in glia, but not neurons, leads to the accumulation of lipid droplets. Furthermore, inactivation of ADAM17 in human iPSC-derived microglia similarly induces aberrant lipid droplet accumulation and mitochondrial reactive oxygen species generation (137), indicating that comparable processes in which TNF works not as an inflammatory trigger but as a trophic survival factor (137) may also be involved in the mammalian retina.

Benoot et al. evaluated the numerous contradictory findings of TNFα application in lung cancer (138), bringing to our awareness the varied functions of various TNF family members and the positive impacts of TNF signaling. Modern genomic, transcriptomic, and proteomic techniques are useful for identifying signaling events and molecules in signal transduction (139, 140). Tanzer et al. (141) discussed in detail how modern proteomic approaches offer a novel perspective on TNF signaling.

Currently, the precise mechanisms of TNFα synthesis remain obscure. Future investigation of TNFα signaling requires the power of new technology, and the potential protective function of TNFα merits greater consideration.

#### 2.1.4 TNFα and microglia-Müller glia interaction

Müller glia exposed to activated microglia modify the expression of a variety of signaling molecules, including (1) elevation of growth factors such as GDNF and leukemia inhibitory factor (LIF), (2) enhanced proinflammatory factor production, and (3) overexpression of chemokines and adhesion proteins (142). TNFα is the most prevalent cytokine produced by reactive microglia, and it stimulates LIF expression in Müller glia in a p38MAPK-dependent manner. Inhibition of p38 MAPK activity lowered LIF expression and accelerated PR mortality in light-damaged retinas (143), similar to previous reports that TNFα prevents cell death by activating the JAK/STAT3 pathway through the IL-6 receptor (144). When TNFα stimuli engage previously activated Müller glia, however, inducible cytokines consisting of more proinflammatory cytokines (TNFα, iNOS, IL-6) and less LIF are produced (145). In other words, depending on the type and degree of stimuli, Müller glia activation generates both neuroprotective and proinflammatory responses, and Müller glia under continuous stimulation are likely to exhibit a detrimental phenotype.

### 2.2 TLR signaling

Toll-like receptors (TLRs) are a class of pattern recognition receptors (PRRs) responsible for identifying pathogen-associated molecular patterns (PAMPs) and DAMPs and mediating immune responses; the generation of PAMPs or DAMPs prompts pathogen invasion or tissue injury. TLR expression is conserved among species, and to date, 10 TLRs (TLR1–10) in humans and 12 TLRs (TLR1–9 and TLR11–13) in mice have been described. TLRs are predominantly but not exclusively expressed on immune cells (146–148).

TLRs serve as the first line of defense for the innate immune system. Upon recognition of DAMPs or PAMPs, TLRs dimerize and initiate recruitment of Toll/IL-1 receptor (TIR) domain-containing adaptor molecules, including myeloid differentiation primary response 88 (MyD88), TIR-domain-containing adaptor-inducing interferon-β (TRIF), MyD88 adaptor-like protein (Mal), and TRIF-related adaptor molecule (TRAM), thereby initiating intracellular signaling cascades: the MyD88- or TRIF-dependent pathways (146). TLR activation facilitates the transduction of NF-κB and MAPK (149–151), as well as the release of proinflammatory cytokines (TNFα, IL-6, IL-1β, and IFNβ), chemokines, and cluster of differentiation 80 (CD80), CD86, CD40, and major histocompatibility complex class II (146).

Activation of TLR signaling has been shown to worsen inflammation and accelerate the course of RP (77, 152, 153). Microglia highly express TLRs (154), and TLR activation in the retina facilitates microglial activation and infiltration (77, 155). Moreover, microglia in the rd1 retina undergo RIP1/RIP3-dependent necroptosis mediated by TLR4 activation, which amplifies retinal inflammation and destruction with large amounts of proinflammatory cytokines (TNFα and CCL2) (152).

#### 2.2.1 DAMPs activate TLRs in RP

High-mobility group box-1 (HMGB1) is a proinflammatory factor and DAMP released by dying cells or activated macrophages that mediates the immune response *via* PRRs (156–158). HMGB1 stimulates an inflammatory response in diabetic retinopathy through TLR4/NF-κB signaling (159).

Increased levels of HMGB1 were detected in the vitreous of patients with RP, along with the presence of necrotic enlarged cone cells (53). In cultured cone-like 661W cells, recombinant HMGB1 treatment induces apoptosis and upregulates the expression of IL-6 and TNFα (160), and external HMGB1 induces retinal ganglion cell death *via* TLR2/4 signaling (161), whereas HMGB1 inhibition or neutralization attenuates the inflammatory response and promotes retinal neuron survival (162, 163).

#### 2.2.2 TLR blockade

Upregulation of Tlr2, Il1b, Myd88 and Tirap was found in RP model rd10 and P23H mice, demonstrating TLR activation involvement in RP-associated retinal degeneration. Genetic deletion of TLR2 alleviated PR loss and vision impairment in both models (76). Similarly, in a light-induced retinal degeneration model, genetic TLR4 deletion reduced retinal inflammation and degeneration (77). Minocycline is an effective microglial inhibitor. In inherited and induced RP models, minocycline administration decreased microglial infiltration and proinflammatory molecule expression and promoted PR survival and functional retention (78–82). Minocycline treatment suppresses MAPK and NF-κB signaling in LPS-stimulated microglia (164), and it has been ascertained that minocycline prevents microglial activation by inhibiting TLR2 (165, 166) and TLR4 (167) signaling.

#### 2.2.3 MyD88

Most TLRs (except for TLR3) use MyD88 as a downstream adaptor protein; moreover, MyD88 is a component of the IL-1R signaling cascade (168, 169). MyD88 features a death domain and a TIR domain. Upon TLR/IL-1R ligation, MyD88 is recruited to the receptor and interacts with IRAK2/4 through their death domains, which activates NF-κB, activator protein-1, and interferon regulatory factors (169).

MyD88 KO mice display attenuated immune responses and are unable to produce normal levels of inflammatory cytokines (170). This diminished immune response preserved PR survival and retinal function during degeneration in rd1 mice lacking MyD88 (83). Similarly, pharmacologic inhibition of MyD88 in rd10 mice with MyD88 inhibitor peptide reduced PR apoptosis and improved rod-related function; treatment also lowered the number of microglia in the PR layer and increased microglia/macrophage expression of the neuroprotective marker Arg1 (84). Further proteomic analysis demonstrated that treatment with such MyD88 inhibitor peptides boosted crystalline expression, suggesting that MyD88 inhibition may also enhance intrinsic tissue-protective mechanisms (85).

#### 2.2.4 AMWAP

Activated microglia/macrophage WAP domain protein (AMWAP), secreted by reactive microglia, is a hallmark of microglial activation. While AMWAP overexpression in microglia lowers the production of proinflammatory factors such as IL-6, iNOS, CCL2, CASP11, and TNFα, extracellular AMWAP endocytosed by microglia inhibits TLR2- and TLR4-induced NF-κB translocation by preventing IRAK-1 and IκBα proteolysis (86). AMWAP administration lowers the apoptosis of 661w cone-like cells treated with microglia-conditioned medium (86), indicating that AMWAP is a potential self-modulator of TLR signaling in microglia.

The TLR signaling pathway plays a fundamental role in inflammatory and immune responses. Molecules released from injured neurons induce an intracellular signaling cascade through TLR/MyD88, contributing to further retinal damage. Blockage of TLR/MyD88 alleviates RP by reducing inflammatory responses and enhancing protective effects.

### 2.3 NLRP3 inflammasome activation

Inflammasomes are cytosolic multiprotein complexes that facilitate the release of mature IL-1β, IL-18, and cleaved caspase-1. The intracellular PRRs, NOD-like receptors (NLRs), are important components of the inflammasome complex. Some NLRs oligomerize upon activation to form multiprotein complexes that function as caspase-1-activating scaffolds (171). NLRP3 is the most well-studied NLR; NLRP3 inflammasome assembly requires two signals: a priming signal that activates NF-κB, followed by transcription of NLRP3, pro-IL-1β, and pro-IL-18. A second activation signal facilitates the recruitment and oligomerization of NLRP3, adaptor protein ASC (apoptosis-associated speck-like protein containing a CARD), and pro-caspase-1. Once the inflammasome is assembled, it stimulates pro-caspase-1 self-cleavage and activation, and cleaved caspase-1 catalyzes pro-IL-1β and pro-IL-18 maturation and induces the release of their mature forms. The recognition of DAMPs or PAMPs that act through PRRs such as TLRs or cytokines that act through particular receptors (TNFR, IL-1R) exemplifies the priming signal. The activation signal encompasses a wide range of stimuli, including ion flux (K+, Cl-, Ca2+), lysosomal instability, mitochondrial dysfunction, reactive oxygen species generation, and trans-Golgi disassembly, with K+ efflux being the upstream event in almost all NLRP3 activations (172, 173).

Inflammasome activation initiates the host’s defense response to endogenous or external damaging stimuli and aids in homeostasis maintenance. Nevertheless, chronic inflammasome activation and the subsequent overproduction of caspase-1, IL-1β, and IL-18 can be detrimental.

Canine models of RP upregulate NLRP3 inflammasome-related genes (26). NLRP3 was detected in cone PRs and one-third of reactive microglia in P23H rhodopsin mutant retinas, which also upregulates the expression of mature IL-1β and IL-18, as well as cleaved caspase-1, indicating inflammasome activation during retinal degeneration. In rd10 mice, administration of the antioxidant N-acetylcysteine prevented PR loss and suppressed inflammatory factors and microglial activation (24). Studies conducted on P23H mice demonstrated that N-acetylcysteine lowered NLRP3 expression by 50% and decreased microglial infiltration, hence improving cone survival and retinal function (87).

#### 2.3.1 P2X7R

The purinergic receptor P2X7R is an adenosine triphosphate (ATP)-gated ion channel and a well-known inflammasome activator that can enhance the expression of the NLRP3 inflammasome in microglia (174). By inducing K+ efflux, ATP-mediated P2X7R activation promotes NLRP3 inflammasome activation (173).

ATP is abundant in PRs as an energy source and neurotransmitter. During retinal degeneration, ATP leaches from dying PRs and activates P2X7R (175). Intravitreal injection of PPADS (pyridoxal-phosphate-6-azophenyl-2’,4’-disulfonic acid), a purinergic antagonist, lowers PR loss in rd1 mice (88). In contrast, intravitreal administration of ATP to WT (wild-type) retinas induces PR degeneration similar to that in the P23H RP model (176), whereas treatment with the selective P2X7R inhibitor BBG (Brilliant Blue G) protects against this ATP-mediated PR apoptosis (177). BBG therapy also reduced inflammasome components (NLRP3, cleaved caspase-1 and mature IL-1β proteins) in P23H retinas (87).

In the absence of extracellular ATP, P2X7R functions as a scavenger receptor that governs microglial clearance of extracellular debris, whereas P2X7R overactivation triggers NLRP3 inflammasome activation by provoking lysosomal instability. Lowering extracellular ATP levels may have the dual benefit of enhancing phagocytosis while decreasing inflammation (178).

#### 2.3.2 IL-1β

IL-1β is a key product of NLRP3 inflammasome activation and a potent immunomodulation factor that orchestrates inflammatory and host defense responses (172, 179). IL-1β signals through IL-1R1. IL-1β binding to IL-1R1 stimulates pathways such as NF-κB, p38, JNKs, ERKs, and MAPKs, facilitating inflammatory cell recruitment and local/systemic inflammatory responses (180). Appropriate IL-1β/IL-1R1 signaling is required for a host’s defensive response to infections, whereas excessive IL-1β signaling is seen in a variety of hereditary and nonhereditary autoinflammatory disorders. IL-1β activity is endogenously regulated by IL-1R2 and IL-1Ra; IL-1R2 is a decoy receptor that sequesters the IL-1β signal, while IL-1Ra blocks IL-1β by competitively binding to IL-1R1 (180).

Intravitreal delivery of exogenous IL-1β triggered an immediate inflammatory response in the retina, including leukocyte recruitment and BRB destruction (181). However, IL-1β does not trigger PR death directly, as IL-1R1 expression is low in PRs. Through IL-1R1 expressed on Müller glia, IL-1β drives glutamate excitotoxicity-induced rod PR loss. The IL-1β/IL-1R1 signal disrupts the process of glutamate conversion into glutamine in Müller glia, resulting in an increased intracellular glutamate concentration, and upregulates xCT (the core subunit of the cystine/glutamate transporter system xc-) expression, which facilitates glutamate release into the extracellular space. Furthermore, IL-1β upregulates the expression of the ionotropic glutamate receptor in retinal neurons, which may increase neuronal vulnerability to glutamate excitotoxicity (182). Infiltrating microglia in the rd10 retina upregulate the expression of IL-1β (19) and block IL-1β signaling using anakinra, a commercially available recombinant IL-1Ra that is fully active in blocking IL-1R1 (183), which reduces PR apoptosis and preserves outer nuclear layer thickness in rd10 animals (19). In contrast, Todd et al. (184) demonstrated that IL-1β expressed by reactive microglia provides neuroprotection *via* IL-1R1 expressed on astrocytes in another mouse model of NMDA-induced retinal degeneration. Despite the use of different models, we were able to determine that IL-1β acts on the surface receptors of distinct glial cells and has varying effects on PR survival.

### 2.4 Chemokine signaling

#### 2.4.1 CX3CL1/CX3CR1

CX3CR1 expression in the central nervous system (CNS) is considered to be restricted to microglia, and the expression of its sole ligand, CX3CL1 (also known as fractalkine), is confined to certain neurons (185). CX3CL1/CX3CR1 signaling facilitates the interaction between neurons and glia and plays a vital role in CNS neuroinflammation (186, 187).

CX3CL1/CX3CR1 signaling contributes to normal microglial and PR function. CX3CR1 signaling governs the dynamic activity of retinal microglia (188). Microglial ablation and repopulation in the mouse retina have shown that microglial recruitment is regulated by CX3CL1/CX3CR1 signaling (40), and Müller glia augment microglial migration and infiltration by increasing CX3CL1 secretion and microglial CX3CR1 expression (68). In addition, CX3CR1 signaling is required for retinal neuron growth, as CX3CR1-deficient retinas have shorter outer segments and diminished cone-related retinal function (189).

CX3CL1/CX3CR1 signaling affects microglial homeostasis by modulating the inflammatory response and phagocytosis. Increasing CX3CL1/CX3CR1 signaling in RP retinas could be beneficial. CX3CR1 deficiency impairs microglial phagocytic clearance of neurotoxic species. Reportedly, CX3CL1 signaling enhances microglial erythrophagocytosis through the CD163/HO-1 axis (190), whereas CX3CR1 KO weakens microglial phagocytosis to β-amyloid and mediates lysosomal dysfunction, resulting in an escalation of neuroinflammation due to β-amyloid accumulation (191).

CX3CR1 deficiency enhances the inflammatory response of microglia. CX3CR1-deficient microglia exhibit greater neurotoxicity (192), and CX3CR1-deficient microglia have an elevated amount of surface P2X7R, which increases IL-1β maturation and release (193). CX3CR1 deletion in microglia-like cells generated from human iPSCs induced enhanced inflammatory responses to LPS stimuli and phagocytic activity to fluorescent beads (194). Loss of CX3CR1 signaling in young animals resulted in a microglial transcriptome similar to that of aged mice, with dysregulated expression of genes related to immune function (195).

CX3CL1 expression is downregulated in rd10 retina before the onset of primary rod degeneration (196), and CX3CR1 KO in rd10 mice increases microglial infiltration and phagocytosis, as well as the generation of pro-inflammatory cytokines, which accelerates PR loss (82, 89), whereas exogenous CX3CL1 supplementation preserves morphology and function (89). CX3CL1 has been shown to deactivate microglia by blocking the NF-κB pathway and activating the Nrf2 pathway (197). A norgestrel-supplemented diet protected rd10 retinas from PR degeneration, and this protection was achieved by the upregulation of CX3CL1/CX3CR1 signaling and the reduction of proinflammatory cytokine production (91, 92).

Recent work by Wang et al. demonstrated that overexpression of soluble CX3CL1 *via* AAV8 prolongs cone survival and improves cone-related visual function in RP model rd1 and rd10 mice. This therapeutic effect is restricted to cone PRs, has no effect on microglial activity or inflammatory factor levels and is not even dependent on the presence of a normal number of microglia (90). In light of this, further research is needed to determine whether CX3CL1 action in the retina is limited to microglia or whether other pathways exist.

#### 2.4.2 CCL2/CCR2

Part of the evidence suggests that CCL2/CCR2 signaling is detrimental, as inhibition of CCL2/CCR2 signaling attenuates microglial activity and degeneration in RP (95–97). CCL2 is highly expressed by stressed PRs, activated microglia, and Müller glia in degenerating retina (95, 198, 199). By binding to its receptor, CCR2, which is expressed on peripheral mononuclear phagocytes, mediates the influx of circulating monocytes into inflamed retinas (200). Using fluorescent protein-labeled *Mertk ^(-/-)^ Cx3cr1 ^(GFP/+)^ Ccr2 ^(RFP/+)^* mice, Kohno H and colleagues demonstrated that both minocycline and lecithin-bound iodine (LBI) ameliorate PR death by inhibiting CCL2/CCR2 signaling (93, 94). Meanwhile, constitutive expression of CX3CR1 in the retina represses CCL2 expression and the recruitment of neurotoxic inflammatory CCR2+ monocytes (201).

However, there is evidence that CCL2 signaling may have a protective role in the degradation of RP. In a light-induced mouse model of degeneration, blocking CCL2/CCR2 signaling decreased infiltrating monocytes but had no effect on the rate of retinal thinning (198). Alde-Low EPCs (low aldehyde dehydrogenase activity endothelial progenitor cells) transplantation therapy rescued vasculature and PRs in rd1 mice, and CCL2 secreted by Alde-Low EPCs recruited a subpopulation of monocyte-derived macrophages that highly expressed CCR2 and the neuroprotective factors TGF-β, IGF-1 and IL-10 (202). In brief, induction of CCL2 expression by Alde-Low EPCs in rd1 retinas resulted in the recruitment of neuroprotective macrophages.

It is apparent that CCL2/CCR2 signaling mediates the recruitment of monocyte-derived macrophages in the degenerating retina, but it remains to be determined whether these recruited cells are beneficial or detrimental.

### 2.5 JAK/STAT signaling

The JAK/STAT signaling pathway is a ubiquitously expressed intracellular signal transduction system implicated in a wide range of biological functions. Various ligands, including cytokines, growth hormones, growth factors, and their receptors, can activate the JAK/STAT pathway (203). Briefly, ligand binding to specific receptors induces receptor multimerization and JAK activation, activated JAKs phosphorylate the receptors, activate and phosphorylate their primary substrate STAT, and phosphorylated STAT dimerizes and translocates into the nucleus, where it binds to particular regions to either activate or inhibit the transcription of target genes. Suppressor of cytokine signaling (SOCS) is a negative modulator of JAK/STAT signaling, and its expression is promoted by stimulation of JAK/STAT signaling (203, 204). Numerous studies on the JAK/STAT pathway have revealed its significance in neoplastic and inflammatory disorders (203, 205).

#### 2.5.1 JAK/STAT and microglia-associated inflammation

Expression and activation of STAT proteins are implicated in the plasticity of the retina during embryonic and postnatal stages (206), and mice deficient in SOCS1/STAT1 develop severe ocular illnesses with massive inflammatory cell infiltration (207). STAT signaling plays a central role in the degeneration of the rd10 retina, as evidenced by proteomic profiling (208). Furthermore, activation of JAK/STAT signaling was also observed in the retinas of light-induced and inherited (rd1 and VPP mouse) RP animal models (98, 209).

AG490 is a JAK2-specific inhibitor that suppresses microglial activation and the production of inflammatory factors such as TNFα and IL-6 by reducing STAT3 phosphorylation (210). AG490 induces M2-type microglial polarization by blocking JAK2/STAT3 signaling in acute paraquat exposure-induced microglial activation (211). In light-damaged retinas, AG490 treatment decreased JAK and STAT phosphorylation as well as PR apoptosis (98).

Olfactory ensheathing cell (OEC) transplantation improved retinal function in RCS rats. OEC treatment dramatically reduced active resident microglia/infiltrated macrophages and the release of proinflammatory cytokines while increasing anti-inflammatory cytokines in the transplantation area. This neuroprotection appears to be mediated in part by increased SOCS3 expression and decreased JAK2/STAT3 activity. Coculture of OECs with the BV2 microglial cell line revealed a shift in microglial cytokine release toward an anti-inflammatory pattern (99). According to the literature, SOCS3-deficient microglia display increased phagocytic activity (212), whereas elevated SOCS3 expression in microglia decreases GM-CSF/IFN-γ-driven inflammatory responses by blocking the activities of JAK1 and JAK2 through its KIR domain (213). In addition, increasing SOCS1 signaling with SOCS1-KIR, a SOCS1 mimetic peptide, suppressed the recruitment of inflammatory cells into the retina and stimulated IL-10 production (214).

Multiple jakinibs (JAK inhibitors) are approved for the clinical management of malignancy, rheumatic, lymphoproliferative, and inflammatory diseases, and most recently, coronavirus disease 2019 (205), but their efficacy in the treatment of RP has not been evaluated.

#### 2.5.2 JAK/STAT and Müller glial neuroprotection

Activation of JAK/STAT signaling has a protective effect on the RP retina. pMSC-derived retinal progenitor cell transplantation increased PR preservation in rd12 mice, and this protection was partially mediated by activation of the JAK/STAT pathway (100).

Application of ciliary neurotrophic factor (CNTF) in RP preclinical research has gained significant neuroprotection and has been employed in clinical trials (101, 215, 216).. CNTF therapy enhances the protective properties of Müller glia through LIF/gp130/STAT3 signaling, thereby preventing retinal degeneration. CNTF treatment elevates the expression of LIF and endothelin 2 (Edn2) (102), and LIF is essential for CNTF-elicited STAT3 activation (217). LIF belongs to the IL-6 cytokine family and signals through the gp130 receptor. In a mouse model of light-induced retinal degeneration, intravitreal delivery of LIF improved PR survival and retinal function by activating STAT3 in Müller glia and PR (103). Stressed PRs secrete signal molecules such as Edn2 and H2O2 that facilitate LIF induction in Müller glia; Edn2 triggers LIF transduction by binding to endothelin receptor B (Ednrb) localized to Müller glia; and H2O2 increases LIF transcript levels by stabilizing LIF mRNA *via* ILF3 (interleukin enhancer binding factor 3) (98, 218–220). LIF deficiency or Ednrb antagonism diminishes JAK/STAT activation and the amount of reactive Müller glia, resulting in accelerated degeneration; in contrast, LIF supplementation or Ednrb agonism improves PR survival in degenerating retina (218, 221).

Deletion of gp130 in either Müller glia or rod PRs severely dampened the activation of CNTF-triggered signaling as well as PR rescue (102), and when Müller glia were ablated, LIF no longer provided protection (222). However, other research suggests that gp130 deficiency in Müller glia decreases STAT3 phosphorylation but does not weaken the neuroprotection of exogenous LIF (223) because gp130 activation in PR presumably mediates a cell-autonomous protective mechanism with a general protective role independent of pathological stimulus (223, 224).

Modulation of JAK/STAT signaling results in contrary immunomodulatory effects in different retinal components. On the one hand, inhibition of JAK2/STAT3 in microglia contributes to inflammation mitigation. On the other hand, LIF-induced STAT3 signaling in Müller glia favors neuroprotection, which seems to be an endogenous protective mechanism. We speculate that this paradoxical outcome involves crosstalk between retinal microglia and Müller glia, which is not yet fully understood. Phosphorylated JAK also activates PI3K, so there may be synergy between JAK/STAT signaling and other pathways.

## 3 Epigenetic modulation in inflammation suppression

Epigenetic modifications, which include DNA methylation, histone modification, and noncoding RNAs, refer to changes in gene expression patterns without altering the genomic DNA sequence (225). Epigenetic modifications are implicated in aspects of individual growth and disease development, including gene expression, cell proliferation and differentiation, misfolded protein response, and cytoskeletal dynamics (226). Although the concept of curing diseases through epigenetic regulation is relatively new, it has demonstrated considerable therapeutic potential in research on cancer, autoimmune diseases, endocrine diseases, congenital disease and many others (227, 228). Epigenetic changes contribute to the development of RP, and remarkable progress has been made in the treatment of RP with epigenetic modification therapies.

### 3.1 Histone acetylation and methylation

Histone acetylation and methylation are the two most well-studied types of histone modification, with acetylation typically resulting in increased gene expression and methylation being related to either increased or decreased gene transcription. Histone acetylation is regulated by histone acetyltransferases and histone deacetylases (HDACs), while histone methylation is regulated by lysine methyltransferases and arginine methyltransferases and histone demethylation by histone demethylases. Enzymes that add or remove epigenetic marks on histones are known as “writers” and “erasers.” In addition, there are “readers” containing bromodomains, chromodomains, or Tudor domains that are able to decipher histone codes (229).

RP retinas exhibit excessive HDAC activity (104, 105, 230), and HDAC inhibition delays retinal degeneration in RP animal models (rd1 and rd10 mice and zebrafish) (104–107). In rd10 mice, the HDAC inhibitor romidepsin prevented rod degeneration and enhanced retinal function. Two molecular mechanisms contribute to this neuroprotective effect. First, by acting on histone targets in PRs, increasing chromatin accessibility and upregulating neuroprotective genes, and second, by acting on nonhistone targets in microglia and resident and invading immune cells, it suppresses inflammatory gene transcription and inflammation (108).

Microglial activity is related to histone methylation levels. LPS-activated microglia increase HDAC expression, which is accompanied by an increase in inflammatory gene expression (231). HDAC inhibition or knockdown promotes a protective microglial phenotype and reduces neuroinflammation (232–234).

Valproic acid is an HDAC inhibitor that reduces PR degeneration in rd1 and P23H RP models (109, 110). Valproic acid increases the expression of STAT1 by inhibiting HDAC3 expression; subsequently, acetylated STAT1 forms a complex with nuclear NF-κB p65, preventing NF-κB p65 DNA-binding activity (235).

Moreover, suppression of the “read” (bind) behavior to histone acetylation marks of bromodomain and extraterminal domain proteins by JQ1 ameliorated PR degeneration and maintained electroretinographic function in rd10 mice. This protection seems to be partially mediated by the inhibition of retinal microglial proliferation, migration, and cytokine production (111).

Several studies, including our previous report, have reported altered histone methylation in RP retinas (112, 236, 237). Lysine demethylase 1 inhibition attenuated PR degeneration in rd10 mice, in part by inhibiting microglial-related inflammation (108). DZNep (3-deazaneplanocin A) specifically inhibits Ezh2 (H3K27 trimethyltransferase) and mediates neuroprotective effects in rd1 mice by inhibiting H3K27me3 deposition (112). Ezh2 reportedly mediates TLR-induced inflammatory gene expression (238) and activation of multiple types of inflammasomes in microglia (239), hence promoting microglial-related pathologies.

### 3.2 MicroRNA

MicroRNAs (miRNAs) are small noncoding RNAs that modify gene expression post-transcriptionally by targeting messenger RNAs, long noncoding RNAs, and pseudogenes and circular RNAs. MiRNAs can be packed into exosomes or microvesicles to perform long-distance cell-to-cell communication. MiRNAs play a critical role in gene expression modulation and are therefore interesting candidates for the development of biomarkers and therapeutic targets (240). Throughout development, miRNAs are required for retinal neuron differentiation (241, 242). Dysregulated miRNAs were found in the retinas of mouse and canine models of RP (243, 244), indicating the involvement of miRNAs in the etiology of RP.

MiRNAs regulate microglial phenotypes, as evidenced by various studies on retinal and neurodegenerative disorders (245, 246). Inhibition of miR-6937-5p preserved the outer nuclear layer thickness and promoted the ERG wave response in rd10 mice (113), and AAV-miR-204 attenuated retinal degeneration in two different mouse models. By downregulating microglial activation and PR mortality, miR-204 alters the expression profiles of transgenic retinas toward those of healthy retinas (114). In addition, miR-223 is required for the regulation of microglial inflammation and the maintenance of normal retinal function (247).

### 3.3 DNA methylation and trained immunity: Epigenetic reprogramming of immunity phenotype

DNA methylation refers to the addition of a methyl group to the 5′-carbon of a cytosine (C) ring, resulting in the formation of 5-methylcytosine (5mC), which mainly occurs in the promoter regions. Typically, methylation modifications result in gene repression, and global genomic hypermethylation relates to heterochromatin formation and inhibits transcription (248). Aberrant regulation of DNA methylation results in PR degeneration and neuronal loss in the retina. In the absence of DNA methyltransferase 1, the initiation of PR differentiation is severely hindered (249). In RP retinas, binding sites of several important transcription factors for retinal physiology were hypermethylated (250). The role of DNA methylation in the development of retinitis pigmentosa has been reviewed in detail elsewhere (251) and will not be repeated here.

We argue that trained immunity regulates the microglial phenotype in RP by plasticizing microglial reactivity *via* epigenetic modification.

**Figure 2:**
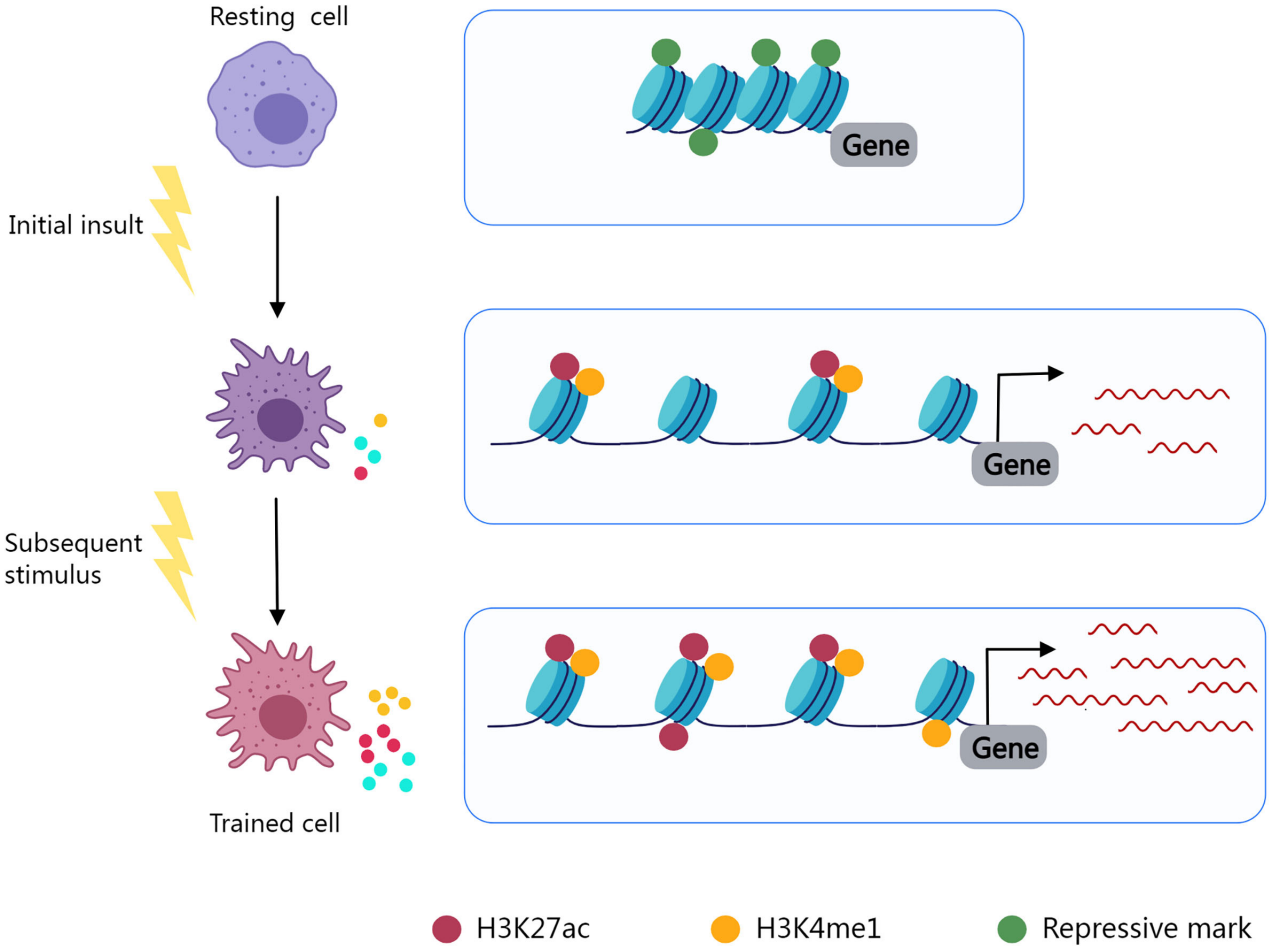
Epigenetic reprogramming determines the immune phenotype of trained cells. Resting cells contain inhibitory epigenetic marks in immune response-related gene regulation areas. Cell activation and epigenetic reprogramming are initiated by an initial insult. Repressive marks fade and epigenetic activating markers (H3K27ac, H3K4me1) are present. Activating markers are partially preserved after stimulus elimination, and trained cells exhibit enhanced immune responses to subsequent stimuli.

Trained immunity, also known as innate immune memory, refers to the phenomenon in which innate immunity modifies its function after an initial insult and reacts more vigorously to subsequent stimuli. Epigenic reprogramming determines the immune phenotype of immune cells and leads to long-lasting functional alterations (252, 253) (**Figure 2**). Using macrophages as an illustration, in the resting state, the promoter regions of inflammatory genes are enriched with repressive epigenetic marks, called epigenetic barriers, to prevent activation in the absence of stimuli. Upon stimulus, repressive epigenetic marks are removed, and activating epigenetic marks are introduced to the promoters and enhancers of specific genes in an attempt to encourage inflammatory molecule synthesis and phagocytosis to eliminate the insult. After stimulus elimination, activating epigenetic marks are partly retained (254). The innate immune system may become overly trained in chronic inflammatory diseases as a result of such mechanisms, resulting in pathological tissue damage.

In the context of neurodegenerative disorders of the CNS, the relationship between trained immunity and microglial phenotype has been discussed (255, 256). Low-dose LPS intraperitoneally administered to mice induced long-lasting innate immune memory in brain microglia and exacerbated Alzheimer’s disease pathology. Activated microglia are enriched with the epigenetic marks H3K4me1 and H3K27ac, which define active enhancers (256). In RP model P23H rats, intraperitoneal injection of low-dose LPS increased microglial activation and the number of infiltrating microglia, as well as elevated the expression levels of several inflammation-related genes (257). In addition to the activation of retinal microglia, elevated levels of serum cytokines show the activation of peripheral immune cells in RP (22, 23). Recent work by Su et al. revealed that monocytes from patients with autosomal recessive RP exhibit a trained-like phenotype. Upon stimulation, these monocytes produce more TNF-α, IL-6, and IL-1β and upregulate inflammatory pathways such as NF-κB (258). Current evidence supports a role for trained immunity in RP pathogenesis by epigenetic reprogramming of microglia and peripheral macrophages to modulate the immune phenotype and trigger an active immune response, although many details remain to be confirmed.

## 4 Gut microbiome and microglial activity

**Figure 3:**
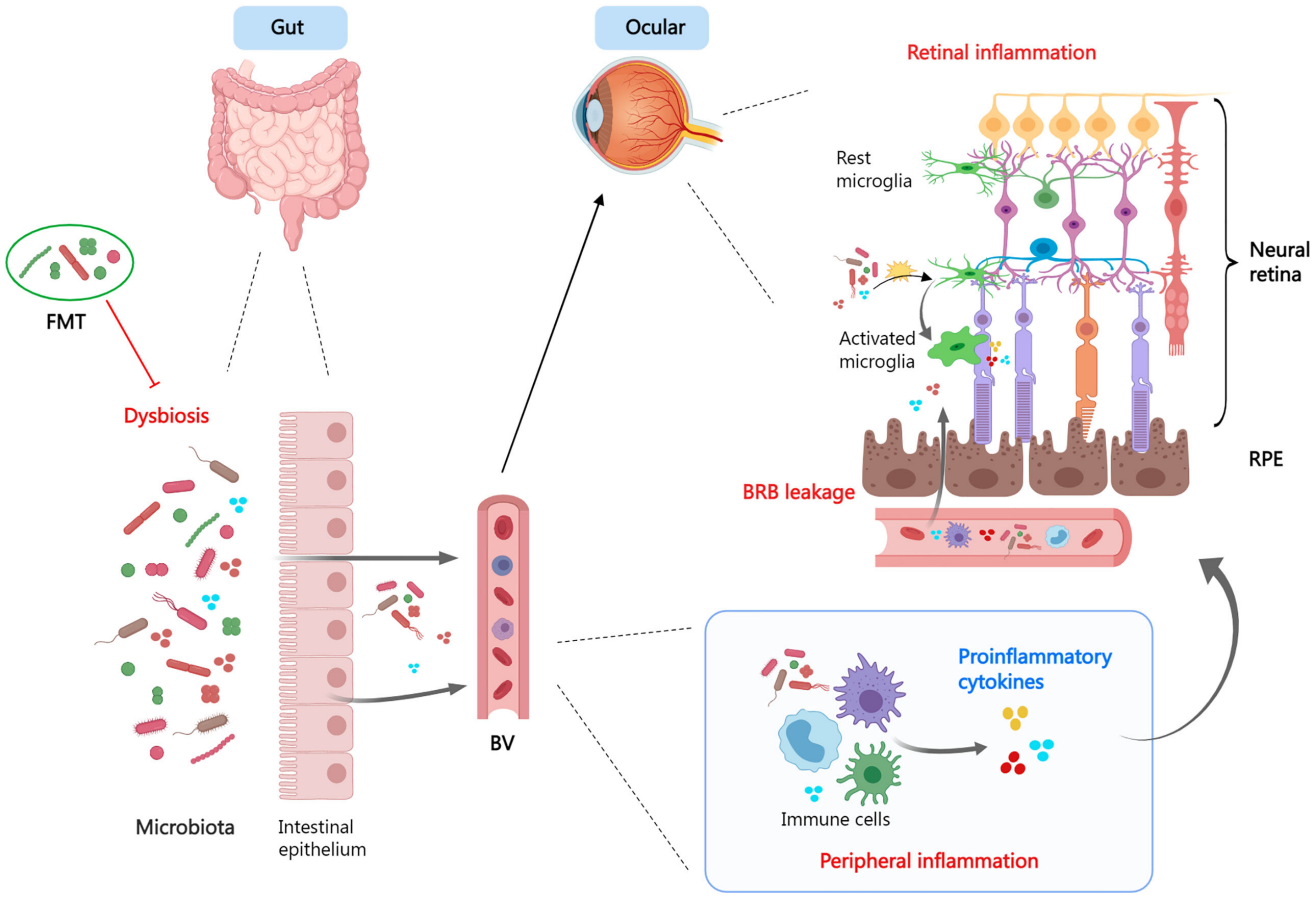
The gut-retina axis. When dysbiosis is present, the microbiota and its derivatives enter the circulation *via* the leaky intestinal mucosa and mediate systemic- and local- (ocular) inflammatory responses. Dysbiosis promotes a pro-inflammatory phenotype of microglia and exacerbates tissue damage. Fecal microbiota transplant is a viable therapeutic strategy. FMT, fecal microbiota transplant; BV, blood vessel; BRB, blood−retina barrier; RPE, retinal pigment epithelium.

The gut microbiome, which resides in the intestinal tract and performs nutrition metabolism, has recently been found to influence the maturation of the immune system. Components and metabolites of microbial cells engage in the modulation of immune recognition and immune tolerance through innate immune receptors on intestinal epithelial cells and influence the function of innate myeloid cells and lymphoid cells through diverse mechanisms (**Figure 3**). In addition, the microbiota’s make-up and function are subject to the innate immune system. Therefore, gut dysbiosis may induce immune system dysregulation and trigger disease emergence (259).

The gut microbiome has been linked to retinal degenerative disorders such as age-related macular degeneration (260) and diabetic retinopathy (261). Using the rd10 RP mouse model, Kutsyr O. et al. (262) related alterations in the composition profiles of the gut microbiome to RP. Compared to healthy mice, the gut microbiome of rd10 mice had reduced ASV richness and α diversity. Rd10 mice, in particular, feature a high proportion of *B. caecimuris*, a species that is uncommon in healthy gut mice, but lack four species (*Rikenella* spp., *Muribaculaceace* spp., *Prevotellaceae* UCG-001 spp., and *Bacilli* spp.) that are common in the healthy gut microbiome (262). The gut microbiome is susceptible to dietary influences. Further research by the same group demonstrated that a short-term high-fat diet significantly modifies the gut flora, enhances retinal oxidative stress and inflammation, and ultimately accelerates the degeneration of the rd10 retina (263). Thus, dysbiosis in the gut contributes to retinal inflammation and constitutes the pathogenesis of RP.

By exchanging the intestinal microbiota (Fecal microbiota transplant, FMT) of young and aged mice, emerging evidence by Parker et al. (264) suggests that the gut microbiome is a modifier of retinal inflammation. Compared to young mice, aged mice exhibit increased systemic and tissue inflammation, as evidenced by elevated serum proinflammatory cytokines (TNFα, IL-6), microglial overactivation in the brain, and C3 accumulation at the RPE/Britch’s membrane interface. Transferring aged donor microbiota to young mice disrupts the intestinal epithelial barrier and triggers inflammation in the retina and brain, whereas transfer of aged mice with young donor microbiota could reverse age-related inflammation (264).

The evidence above supports the “diet-gut microbiome-retina axis” hypothesis in the pathogenesis of RP. Despite the fact that this work is still in its early stages, the gut microbiota is a promising therapeutic target for RP.

## 5 Herbal agents in inflammation suppression

Herbal compounds, or phytochemicals derived from plants, possess a wide range of biological activities and have been explored for the treatment of RP, demonstrating anti-inflammatory properties in RP investigations.

Curcumin is a polyphenolic compound produced from the spice turmeric. Curcumin provided morphological and functional protection in rd1 mice, P23H rats, and an MNU-induced RP model (115, 116, 265, 266). A single vitreous injection of curcumin reduced PR loss in rd1 mice by inhibiting microglial activation and modulating the expression of CCL2, TIMP-1 and VCAM-1 (115).

Lyceum barbarum polysaccharides and zeaxanthin dipalmitate are two main bioactive agents extracted from wolfberry. Lyceum barbarum polysaccharides protects against retinal degeneration by modifying inflammation and apoptosis through the inhibition of NF-κB and HIF-1α expression (117, 118). zeaxanthin dipalmitate acts through several pathways, including STAT3, CCL2 and MAPK, in parallel to inhibiting inflammation in the rd10 retina (119).

Saffron, widely used in traditional Chinese medicine for its anti-inflammatory and antioxidant properties, protects PRs exposed to environmental ATP by blocking P2X7R signaling (120). In P23H rats, saffron administration increased PR survival and functional retention while decreasing vascular disruption (121).

Resveratrol (3,40,5-trihydroxystilbene) is found in chocolate, fruits, and vegetables. Resveratrol treatment inhibited microglia-mediated death of 661W cells *via* downregulation of microglial migratory, phagocytic, and proinflammatory cytokine production (122). Subretinal injection of JC19 (3,4’-diglucosyl resveratrol), a resveratrol prodrug, reduced PR loss and improved functional performance in ERG tests of the rd10 retina. The author speculates that sirtuin1 activation is the underlying mechanism (123).

## 6 Conclusion and future perspectives

A large body of research conducted on the inflammatory processes during RP tries to discover common mechanisms that target multiple RP genotypes and develop appropriate therapeutic options. However, after reviewing the existing literature, we discovered that no single treatment is appropriate for all types of RP, and the application of valproic acid is a prime example, with treatment effects significantly varying between models with different genetic backgrounds and even exhibiting detrimental effects. This raises the prospect that a link between genetics and RP inflammation needs more investigation. To date, genetic mutations remain the only identified risk factor for RP. Different permutations of inheritance pattern, genotype, and the number of mutations lead to variations in the phenotype and pathological progression of RP. Similarly, we anticipate that the multiple phenotypes of inflammatory activation in RP are closely related to the genetic background. Nevertheless, the relationship between genetic background and inflammation is currently unclear due to the lack of corresponding evidence.

Both RP patients and animal models have a more susceptible immune system and are prone to developing inflammation. This abnormal immune system may depend heavily on the genetic background. The gut microbiota play a critical role in the maturation of the innate immune system after birth, and trained immunity is implicated in this process; however, the influence of genetic background on the maturation of the immune system has not been investigated. Therefore, long-term clinical observation and family tracing of the RP population are necessary. What needs to be documented should include, but is not limited to, macroscopic clinical manifestations, structural and functional measurements, and monitoring of local and peripheral inflammation levels. And appropriate follow-up criteria need to be established to ensure consistency of measurements and to obtain usable information.

Inflammation is an important feature of RP, and the present review highlights the role of immunomodulation in RP treatment. There has been significant interest in modulating the inflammatory response as a strategy to treat RP, and an increasing number of studies have proven the effectiveness of immunomodulation in ameliorating and perhaps reversing retinal degeneration. Therapeutic strategies based on immunomodulation are a potential treatment for RP, and deepening the understanding of immune modulation is helpful in establishing suitable therapies. As with immunotherapies already carried out, artificial regulation of immunity will bring inevitable side effects. It is challenging to regulate immunity accurately and to enhance the beneficial effects and minimize the harmful ones concurrently. Many of these specific mechanisms need to be further studied, especially the interactions between these pathways.

## Author contributions

LZ wrote the manuscript and painted the figure. NY and CH reviewed and modified the article. All authors contributed to the article and approved the submitted version.

## Funding

This work was supported by grants from the Science and Technology Department of Sichuan Province (2020YFS0205).

## Conflict of interest

The authors declare that the research was conducted in the absence of any commercial or financial relationships that could be construed as a potential conflict of interest.

## Publisher’s note

All claims expressed in this article are solely those of the authors and do not necessarily represent those of their affiliated organizations, or those of the publisher, the editors and the reviewers. Any product that may be evaluated in this article, or claim that may be made by its manufacturer, is not guaranteed or endorsed by the publisher.
